# Metropolitan Hospital Sunday Fund

**Published:** 1917-12-29

**Authors:** 


### METROPOLITAN HOSPITAL SUNDAY FUND.

### Annual Meeting of Constituents.

### £76,354 DISTRIBUTED.

The annual meeting of constituents of this Fund was held at the Mansion House on Thursday, December 20, the Lord Mayor (the Right Hon. Charles Augustine Hanson), the President and Treasurer of the Fund, in the chair. There was a fair attendance, those present including Sir Charles C. Wakefield, Bart., the Right Hon. Lord Somerleyton, P.C., Sir William Church, Bart., K.C.B., Sir Dyce Duckworth, Bart., Rev. Prebendary Swayne, the Archdeacon of London, the Rev. Prebendary Reynolds, and Right Rev. Mgr. Carton de Wiart.

Letters of regret for absence had been received from the Rev. Prebendary Grose Hodge, Lord Meath, and Mr. Robert Holland-Martin.

### The Annual Report.

The meeting having agreed to accept the annual report as read,

**Sir C. Ernest Tritton, Bart.,** moved its reception and adoption. He said he questioned whether our hospitals had ever before rendered such signal service to the community as they had in the year under review. Their services to our wounded sailors, soldiers, and airmen—gallant fellows who deserved the very best possible treatment—especially their care for the men, women, and little children injured in the dastardly air raids to which London had been subjected, and their never-failing services to the sick poor of this great city, combined to put all its inhabitants under a sense of deep indebtedness to the hospitals, and stir citizens to fresh effort on behalf of those noble institutions. The Report showed that the fund had had a very fairly successful year, for it was distributing this year more than in any year since 1895, when the total included a special legacy of £12,500. He used the phrase "fairly successful" because the Council were not satisfied with what had been done; they wished for still greater efforts, that the claims of the hospitals should be pressed still more strongly upon the people of London. The Council felt intensely indebted to the clergy and ministers of all denominations for what they had done in swelling the contributions to a total £2,218 in excess of that for the previous year. He would earnestly suggest to the clergy and ministers whether it might be possible to adopt a plan for enlisting the practical sympathy not only of regular church-goers and chapel-goers, but of those outside places of worship, for he felt that they also were keenly interested in the hospitals, and would willingly help if afforded the opportunity. The extracts from letters given in the Report showed with what splendid success a scheme of house-to-house visitation had worked. He believed that with an extended organisation there would result an immensely larger Fund, for the people loved the hospitals which were doing such a fine work for those near and dear to them. The hospitals would need help in the future more than ever, owing to the prices of provisions and drugs having risen so greatly. He urged all to put their backs into the Fund with a will, and make it an even greater success. In conclusion he paid a warm tribute to the Secretary of the Fund, Mr. Arnold James, and his assistant Mr. Lander, for their enthusiastic services.

**The Rev. J. Stanway** seconded the resolution with great pleasure. Having worked in three different parts of the Metropolis, he could bear witness to the great value of the Fund to the sick poor, and he felt that the very best should be done to support it. He spoke with great satisfaction of the promptitude with which an appeal directed to the Fund was replied to, either in the form of a grant or a reply of some kind. That was very important, and promptness of reply was not a universal experience in these days.

The resolution was carried unanimously.

**The Rev. W. Russell** moved the re-election of the following gentlemen, who retire in accordance with Law VIII. : The Bishop of London, the Bishop of Stepney, the Great Archimandrite, Rev. Prebendary Eardley-Wilmot, Rev. H. Newell Bate, Rev. Mgr. Carton de Wiart, Rev. Charles Brown, Rev. S. Scott Lidgett, Rev. H. A. Hall, Rev. J. Gough McCormick, Rev. Morris Joseph, the Earl of Meath, Lord George Hamilton, Hon. Sir W. H. Goschen, Hon. Frederick Ponsonby, Sir C. Arthur Pearson, Sir A. Pearce Gould, Andrew Motion, Esq., John Robarts, Esq., and Edward Dent, Esq., and the election of the following gentlemen to fill the vacancies : Rev. Prebendary Grose Hodge, Rev. Austin Thompson, John Latta, Esq., J. E. Moxey, Esq., Dr. W. T. Partridge, and Robert M'Connell, Esq. The good work of the gentlemen who were eligible for re-election was well known and recognised, and he did not doubt that, under such associations, the new members would carry on the same tradition.

**Mr. F. H. Sully** seconded, and the resolution was unanimously adopted.

### Hospital Sunday, 1918.

**The Right Hon. Lord Somerleyton, P.C.,** moved: "That in pursuance of the resolution of the last annual meeting, Hospital Sunday in 1918 be, and is hereby, fixed for June 23." He congratulated the Lord Mayor on the fact that the Fund had reached its best total for many years in the year that he had attained the Chief Magistracy, and shared Sir Ernest Tritton's hope for an even greater prosperity in the future. It was significant that the King's Fund had also distributed more this year than ever before, and he hoped the Saturday Fund would have a similar record. He thought much of the success of the Hospital Sunday Fund was due to the enthusiasm of Sir Ernest Tritton, for he had attended to the financial side of it with his usual ability, and had put into it a great deal of hard work.

**Sir Marcus Samuel, Bart.,** seconded, and the resolution was carried.

**The Ven. the Archdeacon of London** proposed a cordial vote of thanks to the Lord Mayor for presiding at the meeting. This was seconded by the **Rev. Scott Lidgett,** and carried by acclamation.

**The Lord Mayor,** in reply, said he did not think any Lord Mayor could feel a more profound interest in this Fund than he did himself. But a vote of thanks to himself was quite unnecessary, as the obligation and the pleasure were his. He hoped all the cherished ambitions and expectations in regard to the Fund would be realised; he would be satisfied if in the coming year the six-figure line could be attained—£100,000. He would be delighted to place his services, for what they were worth, at the disposal of the Fund, and do what he could to make of it a great success.

